# Neutrophil gelatinase-associated lipocalin monitoring reveals persistent subclinical kidney injury following intraarterial administration of iodinated contrast agents

**DOI:** 10.1038/s41598-022-24169-7

**Published:** 2022-11-14

**Authors:** Alina Scridon, Cristina Somkereki, Tunde Renata Nicoară, Mădălina Oprica, Liliana Demian

**Affiliations:** 1 University of Medicine, Pharmacy, Science and Technology “George Emil Palade” of Târgu Mureș, Târgu Mureș, Romania; 2 Emergency Institute for Cardiovascular Diseases and Transplantation Târgu Mureș, Târgu Mureș, Romania; 3 Physiology Department, University of Medicine, Pharmacy, Science and Technology “George Emil Palade” of Târgu Mureș, 38, Gheorghe Marinescu Street, 540139 Târgu Mureș, Romania

**Keywords:** Biochemistry, Physiology

## Abstract

Clinically overt contrast-induced nephropathy (CIN) is one of the most feared complications in patients exposed to iodinated contrast media and has been extensively studied over the years. Meanwhile, the incidence and evolution of subclinical contrast-induced kidney injury remain elusive. With the continuous increase in the number of patients that are repeatedly exposed to contrast media, elucidating these issues is of critical importance. Accordingly, we aimed to evaluate the incidence and the evolution of clinical and subclinical kidney injury in patients exposed to contrast media. A total of 178 patients who underwent elective percutaneous angioplasty procedures were evaluated prospectively. Serum creatinine and neutrophil gelatinase-associated lipocalin (NGAL) levels were evaluated pre-procedurally, 48 h and 1 month after administration of contrast media. The evolution of creatinine and NGAL levels was analyzed at the three time points, and the potential predictors of contrast-induced clinical and subclinical renal injury were evaluated. Clinically overt CIN occurred in 10 (5.6%) patients. Baseline serum creatinine and the volume of contrast media were the only independent predictors of CIN and in all 10 patients creatinine levels returned to baseline by 1 month (p = 0.32). Subclinical contrast-induced kidney injury was much more common, affecting 32 (17.9%) patients, was only predicted by the baseline serum creatinine, and persisted in 53.1% of patients after 1 month. This study showed that whereas clinically overt CIN is rather rare and regressive, subclinical contrast-induced kidney injury is considerably more frequent, affecting almost 18% of patients that receive intraarterial contrast media. More importantly, subclinical kidney injury persisted after 1 month in more than 50% of the initially affected patients, who may thus be at increased risk for further renal impairment, particularly if exposed to nephrotoxic agents or repeated administration of contrast media.

## Introduction

In parallel with the major advancements in diagnostic and therapeutic imaging techniques, the number of patients exposed to iodinated contrast agents has tremendously increased over the past decades^1^. One of the most feared complications in these patients is contrast-induced nephropathy (CIN). The use of less nephrotoxic agents has significantly reduced this risk. Yet, the risk of CIN remains considerable (2–25%) in this population, with up to 0.5% of patients requiring dialysis^2^.

Currently, the diagnosis of CIN relies on a relative and/or absolute increase in serum creatinine following contrast administration, reflecting an acute impairment in kidney function^3^. The same criteria, based on altered creatinine levels, suggest that CIN is most commonly a transient process that is usually followed by full restoration of kidney function within 7–14 days. However, the exact effects of contrast administration on the kidneys remain incompletely elucidated, particularly over the long term.

Serum creatinine alone provides only a rough estimation of the impact of contrast agents on the kidneys and is incapable to exclude the persistence of a degree of subclinical kidney injury over the long term^4^. Pathophysiologically, persistence of at least a certain degree of tubular damage following contrast administration seems highly plausible. The occurrence of CIN has been linked mechanistically to vasoconstriction, followed by renal hypoperfusion and hypoxia, cytotoxicity caused by increased local production of reactive oxygen species, and direct tubular toxicity, characterized by vacuolization and necrosis of kidney tubular cells^5^. Episodes of ischemia–reperfusion similar to those seen in CIN have also been related to subsequent loss of peritubular capillaries and progressive tubular fibrosis^6^. Together, these observations strongly suggest that renal injury associated with contrast administration may not be entirely transient and that biomarkers more sensitive than creatinine may be needed to fully understand the impact of contrast agents on the kidneys.

Neutrophil gelatinase-associated lipocalin (NGAL), a glycoprotein that is rapidly released into the bloodstream in response to renal tubular injury^7^, appears to be a promising candidate in this regard. Unlike serum creatinine, which provides a rather late and strictly functional reflection of renal injury, NGAL is an early marker of tubular damage that can unmask the presence and the evolution of renal injury even in the absence of a significant functional impairement^4^. With the widespread use of procedures that rely on contrast administration, many patients are likely to receive repeated doses of contrast agents throughout their lives. If contrast-induced kidney injury proves to be persistent, even at a subclinical level, such an effect could become highly relevant in the following decades.

Accordingly, in the present study, we aimed (1) to evaluate the incidence of clinical and subclinical kidney injury in patients exposed to iodinated contrast agents and (2) to assess the evolution of clinical/subclinical kidney injury in these patients over the long term.

## Methods

### Study population

Consecutive patients who underwent elective angioplasty procedures in our center between January 2020 and November 2021 were evaluated in a prospective observational study. All patients included in the study were ≥ 18 years of age, were admitted to hospital for an elective angioplasty procedure, and had an estimated glomerular filtration rate (eGFR) > 30 mL/min/1.73 m^2^. Exclusion criteria included acute severe conditions, regardless of their nature, ongoing non-steroidal antiinflammatory or antibiotic therapy, exposure to contrast media over the past 6 months, dialysis or history of kidney transplantation, multiple myeloma, and lymphoplasmacytic lymphoma. The research protocol complied with the Declaration of Helsinki and was approved by the local Ethics Committees of the Emergency Institute for Cardiovascular Diseases and Transplantation Târgu Mureș (approval number 7545) and of the University of Medicine, Pharmacy, Science and Technology “George Emil Palade” of Târgu Mureș (approval number 230). All patients gave written informed consent to participate in the study.

### Evaluated parameters

#### Baseline evaluation

Age, gender, body mass index, left ventricular ejection fraction assessed by transthoracic echocardiography, associated conditions (i.e., arterial hypertension, diabetes mellitus, heart failure), and ongoing therapy with potentially nephrotoxic (i.e., angiotensin converting enzyme inhibitors, angiotensin II receptor blockers, diuretics, metformin) and/or nephroprotective (i.e., methylxanthines, statins, ascorbic acid, N-acetyl cysteine, dihydropyridine calcium channel blockers) drugs were recorded for each patient on admission. Arterial hypertension was considered present if a diagnosis of hypertension was present in the patient’s medical records, was established during hospital stay, in accordance with current guidelines, or if the patient was under ongoing antihypertensive therapy. Pre-procedural hydration status was evaluated in all patients based on the maximum inferior vena cava diameter measured by echocardiography in subcostal 4-chamber view. Venous blood samples were collected from each patient prior to the angioplasty procedure and total blood count, hemoglobin, plasma glucose and lipids, total protein and albumin, and uric acid levels were evaluated using standardized laboratory tests. Serum creatinine was measured using a modified buffered kinetic Jaffe reaction and eGFR was calculated using the Modification of Diet in Renal Disease (MDRD) equation. Serum levels of NGAL were measured using an enzyme-linked immunosorbent assay (Biovendor, Czech Republic; intra- and inter-assay variation coefficients 7.0% and 9.8%, respectively) on the Elisa Dynex DSX fully automated Elisa analyzer (DYNEX Technologies, Inc.; Chantilly, VA). The volume and type (i.e., iomeprol or ioversol) of contrast agent administered were also recorded.

#### Evaluation at 48 h and at 1 month following the angioplasty procedure

The Mehran score^8^ was calculated for each patient 24 h after the angioplasty procedure. Administration of potentially nephrotoxic and/or nephroprotective drugs, as listed above, during the 48 h after the angioplasty procedure was recorded for each patient. Occurrence of death, cardiogenic shock, acute pulmonary edema, the need for dialysis and/or positive inotrope agents during the 48 h after the procedure, and the length of hospital stay were also recorded. A second venous blood sample was collected from each patient 48 h after the angioplasty procedure and the serum levels of creatinine, total protein and albumin, uric acid, sodium, and NGAL were measured as described above. The eGFR was calculated using the MDRD equation.

Administration of potentially nephrotoxic and/or nephroprotective drugs and the occurrence of death, interventions requiring repeated contrast administration, hospitalizations, and the need for dialysis during the first month following the angioplasty procedure were also recorded. A third blood sample was collected from each patient 30 days after the angioplasty procedure and serum creatinine, total protein and albumin, uric acid, and NGAL levels were measured as described above. The eGFR was calculated using the MDRD equation.

### Outcomes definitions

In accordance with current recommendations^9^, CIN was defined as an increase in serum creatinine by at least 25% 48 h following contrast administration *vs*. baseline. Early subclinical kidney injury was defined as an increase in NGAL by at least 25% 48 h following contrast administration compared to baseline. Kidney dysfunction/injury was considered persistent if serum creatinine or NGAL levels, respectively, remained at least 25% higher 1 month following contrast administration compared to baseline in patients in whom early CIN or subclinical kidney injury was initially detected.

### Statistical analysis

Continuous variables are presented as mean values ± standard deviation or median and interquartile range, as appropriate. Categorical data are summarized using frequencies and percentages. The occurrence of early (i.e., at 48 h after contrast administration) kidney dysfunction/injury was assessed by comparing renal parameters (i.e., serum creatinine and NGAL) measured at 48 h after contrast administration *vs*. baseline using the paired *t*-test or the Wilcoxon matched-pairs signed-ranks test, as appropriate.

The patients were divided into groups depending on the presence or absence of CIN and on the presence or absence of subclinical kidney injury, respectively, and all parameters were compared between groups using the unpaired *t*-test or the Mann–Whitney *U* test (for continuous variables), or Fisher’s exact test (for categorical data). Logistic regression analysis was used to assess predictors of CIN and of early subclinical kidney injury. The models were adjusted for parameters that differed significantly between the groups. For the continuous variables, the cutoff values were established using receiver operating characteristic analysis. The evolution of kidney dysfunction/injury at 1 month was evaluated in patients who presented CIN or early kidney injury by comparing renal parameters (i.e., serum creatinine and NGAL) measured at 1 month vs. those measured at 48 h after contrast administration using the paired *t*-test or the Wilcoxon matched-pairs signed-ranks test, as appropriate. All tests were two-sided, and a p-value of less than 0.05 was considered statistically significant. All data were computed using MedCalc for Windows, version 12.4.3.0 (MedCalc Software; Ostend, Belgium).

## Results

### Study population characteristics

A total of 178 patients (mean age 63.2 ± 9.2 years; 69.1% male) were included in the present study. Pre-procedural intravenous hydration (saline solution, 1 ml/kg/h, 12 h before and 12 h after the procedure) was used in 35 (19.6%) of the study patients. All patients were evaluated at baseline and at 48-h follow-up and 63 of the 178 study patients completed the 1-month follow-up. Patients’ characteristics at the three time points are summarized in Table 1.

**Table 1:** Characteristics of patients included in the study.

**Baseline characteristics (n = 178)**	
Age (years)	63.2 ± 9.2
Male gender (n, %)	123 (69.1%)
Body mass index (kg/m^2^)	28.8 ± 4.1
Left ventricular ejection fraction on admission (%)	50.8 ± 9.1
Comorbidities	
Arterial hypertension (n, %)	162 (91.0%)
Diabetes mellitus (n, %)	80 (44.9%)
Heart failure (NYHA class)	2 (2–2)
Chronic kidney disease (n, %)	32 (17.9%)
Ongoing therapy with potentially nephrotoxic drugs	
ACEI (n, %)	116 (65.1%)
ARB (n, %)	39 (21.9%)
Diuretic (n, %)	80 (44.9%)
Metformin (n, %)	43 (24.1%)
Ongoing therapy with potentially nephroprotective drugs	
Methylxanthine (n, %)	0 (0%)
Statin (n, %)	176 (98.8%)
Ascorbic acid (n, %)	0 (0%)
N-acetyl cysteine (n, %)	0 (0%)
Dihydropyridine calcium channel blocker (n, %)	73 (41.0%)
Inferior vena cava diameter (cm)	1.9 ± 0.2
Laboratory parameters on admission	
White blood cells (/mm^3^)	7669 ± 1928
Platelets (/mm^3^)	234,662 ± 67,053
Hemoglobin (g/dL)	13.6 ± 1.6
Glucose (mg/dL)	141.4 ± 63.7
Total cholesterol (mg/dL)	162.0 ± 48.5
Triglycerides (mg/dL)	161.0 ± 101.7
Total protein (g/dL)	66.0 ± 4.8
Albumin (g/dL)	4.3 ± 0.2
Uric acid (mg/dL)	6.2 ± 1.7
Serum creatinine (mg/dL)	1.05 ± 0.36
eGFR (mL/min/1.73 m^2^)	76.31 ± 23.43
NGAL (ng/mL)	75.79 ± 26.46
Contrast agent administered	
Type-iomeprol (n, %)	153 (85.9%)
Volume (mL)	148.5 ± 68.2
Pre-procedural intravenous hydration (n, %)	35 (19.6%)
**Parameters at 48 h after contrast administration (n = 178)**	
Mehran score (points)	4 (1–6)
Therapy with potentially nephrotoxic drugs during the past 48 h	
ACEI (n, %)	114 (64.0%)
ARB (n, %)	43 (24.1%)
Diuretic (n, %)	84 (47.1%)
Metformin (n, %)	43 (24.1%)
Therapy with potentially nephroprotective drugs during the past 48 h	
Methylxanthine (n, %)	0 (0%)
Statin (n, %)	176 (98.8%)
Ascorbic acid (n, %)	0 (0%)
N-acetyl cysteine (n, %)	0 (0%)
Dihydropyridine calcium channel blocker (n, %)	80 (44.9%)
Complications during the past 48 h	
Death (n, %)	0 (0%)
Cardiogenic shock (n, %)	0 (0%)
Acute pulmonary edema (n, %)	0 (0%)
Dialysis (n, %)	0 (0%)
Positive inotrope agents (n, %)	0 (0%)
Length of hospital stay (days)	4 (3–6)
Laboratory parameters 48 h after contrast administration	
Total protein (g/dL)	65.5 ± 5.1
Albumin (g/dL)	4.3 ± 0.3
Uric acid (mg/dL)	6.0 ± 1.6
Serum creatinine (mg/dL)	1.01 ± 0.39
eGFR (mL/min/1.73 m^2^)	80.17 ± 23.49
NGAL (ng/mL)	76.64 ± 39.64
**Parameters 1 month after contrast administration (n = 63)**	
Therapy with potentially nephrotoxic drugs during the past month	
ACEI (n, %)	47 (74.6%)
ARB (n, %)	11 (17.4%)
Diuretic (n, %)	24 (38.0%)
Metformin (n, %)	16 (25.3%)
Therapy with potentially nephroprotective drugs during the past month	
Methylxanthine (n, %)	0 (0%)
Statin (n, %)	60 (95.2%)
Ascorbic acid (n, %)	0 (0%)
N-acetyl cysteine (n, %)	0 (0%)
Dihydropyridine calcium channel blocker (n, %)	32 (50.7%)
Complications during the past month	
Death (n, %)	0 (0%)
Repeated contrast administration (n, %)	0 (0%)
Hospitalization (n, %)	0 (0%)
Dialysis (n, %)	0 (0%)
Laboratory parameters 1 month after contrast administration	
Total protein (g/dL)	70.7 ± 5.9
Albumin (g/dL)	4.5 ± 0.2
Uric acid (mg/dL)	5.7 ± 1.2
Serum creatinine (mg/dL)	0.96 ± 0.24
eGFR (mL/min/1.73 m^2^)	72.6 ± 16.3
NGAL (ng/mL)	79.75 ± 31.91

eGFR was calculated using the Modification of Diet in Renal Disease equation.

Quantitative data are expressed as mean values ± standard deviation or median and interquartile range, as appropriate. Categorical data are expressed as number (percentage).

*ACEI* angiotensin converting enzyme inhibitor, *ARB* angiotensin II receptor blocker, *eGFR* estimated glomerular filtration rate, *NGAL* neutrophil gelatinase-associated lipocalin, *NYHA* New York Heart Association.

### Early (48 h) and late (1 month) post-contrast administration kidney dysfunction/injury

There was no significant difference in serum creatinine levels measured 48 h (1.01 ± 0.39 mg/dL) or 1 month (0.96 ± 0.24 mg/dL) following contrast administration vs. baseline (1.05 ± 0.36; both p > 0.05). Consequently, no significant difference was recorded in eGFR in neither of the two moments (i.e., 48 h and 1 month after contrast administration) vs. baseline (both p > 0.05). The same results were obtained when NGAL levels were assessed comparatively at the three time points (both p > 0.05).

Overall, 10 (5.6%) of the 178 study patients fulfilled the criteria for CIN 48 h after contrast administration and in all of them creatinine levels returned to baseline values by the 1-month follow-up (p = 0.32). Compared to patients who did not develop CIN, those who presented CIN (Table 2) had higher serum creatinine levels (p = 0.03), lower eGFR (p = 0.02), and lower left ventricular ejection fraction (p < 0.01) on admission, were more often anemic (p = 0.001) and on diuretic treatment (RR 29.3 [95%CI 1.6–50.9]; p < 0.001) on admission, received higher volumes of contrast media (p = 0.01), and had longer duration of hospital stay (p < 0.01). However, in the logistic regression analysis, only a baseline serum creatinine > 1.62 mg/dL (and a baseline eGFR ≤ 63 mL/min/1.73  m^2^; Supplementary material) and a volume of contrast agent administered > 180 mL remained independent predictors of CIN (Table 3).

**Table 2:** Characteristics of patients who developed *versus* those who did not develop contrast-induced nephropathy.

Parameter	CIN (n = 10)	No CIN (n = 168)	p-value
**Baseline characteristics**			
Age (years)	66.7 ± 5.5	63.4 ± 9.3	0.49
Male gender (n, %)	8 (80.0%)	115 (68.4%)	0.72
Body mass index (kg/m^2^)	28.3 ± 6.7	29.0 ± 4.0	0.93
Left ventricular ejection fraction on admission (%)	37.5 ± 19.3	51.4 ± 7.8	< 0.01
Comorbidities			
Arterial hypertension (n, %)	10 (100.0%)	152 (90.4%)	0.60
Diabetes mellitus (n, %)	5 (50.0%)	75 (44.6%)	0.75
Heart failure (NYHA class)	2 (1.5–2.5)	2 (2.0–2.0)	0.64
Ongoing therapy with potentially nephrotoxic drugs			
ACEI (n, %)	8 (80.0%)	108 (64.2%)	0.49
ARB (n, %)	2 (20.0%)	37 (22.0%)	1.00
Diuretic (n, %)	10 (100.0%)	70 (41.6%)	< 0.001
Metformin (n, %)	2 (20.0%)	41 (24.4%)	1.00
Ongoing therapy with potentially nephroprotective drugs			
Statin (n, %)	10 (100.0%)	166 (93.2%)	1.00
Dihydropyridine calcium channel blocker (n, %)	2 (20.0%)	71 (42.2%)	0.20
Inferior vena cava diameter (cm)	1.8 ± 0.1	2.0 ± 0.2	0.37
Laboratory parameters on admission			
White blood cells (/mm^3^)	8430 ± 977	7696 ± 1991	0.19
Platelets (/mm^3^)	179,250 ± 95,175	239,053 ± 65,638	0.23
Hemoglobin (g/dL)	13.3 ± 0.9	13.6 ± 1.7	0.62
Anemia (n, %)	8 (80.0%)	45 (26.7%)	0.001
Glucose (mg/dL)	172.7 ± 72.0	140.9 ± 63.1	0.19
Total cholesterol (mg/dL)	148.5 ± 36.1	162.9 ± 49.0	0.70
Triglycerides (mg/dL)	153.0 ± 69.4	156.7 ± 94.1	0.76
Total protein (g/dL)	70.4 ± 4.1	65.6 ± 4.8	0.09
Albumin (g/dL)	4.6 ± 0.4	4.3 ± 0.2	0.19
Uric acid (mg/dL)	7.7 ± 3.2	6.1 ± 1.5	0.39
Serum creatinine (mg/dL)	1.43 ± 0.15	1.02 ± 0.34	0.03
eGFR (mL/min/1.73 m^2^)	58.25 ± 26.84	78.68 ± 23.19	0.02
Pre-procedural intravenous hydration (n, %)	3 (30.0%)	32 (19.0%)	0.41
Contrast agent administered			
Type-iomeprol (n, %)	8 (80.0%)	145 (86.3%)	0.63
Volume (mL)	255.0 ± 108.4	140.0 ± 61.6	0.01
**Parameters at 48 h after contrast administration**			
Mehran score (points)	7 (2.5–13.0)	4 (1.0–6.0)	0.27
Therapy with potentially nephrotoxic drugs during the past 48 h			
ACEI (n, %)	8 (80.0%)	106 (63.0%)	0.33
ARB (n, %)	2 (20.0%)	41 (24.4%)	1.00
Diuretic (n, %)	10 (100.0%)	74 (44.0%)	< 0.001
Metformin (n, %)	2 (20.0%)	41 (24.4%)	1.00
Therapy with potentially nephroprotective drugs during the past 48 h			
Statin (n, %)	10 (100.0%)	166 (93.2%)	1.00
Dihydropyridine calcium channel blocker (n, %)	2 (20.0%)	78 (46.4%)	0.19
Length of hospital stay (days)	10.0 ± 3.5	4.7 ± 2.1	< 0.01
Laboratory parameters 48 h after contrast administration			
Total protein (g/dL)	65.5 ± 8.4	65.3 ± 5.1	0.82
Albumin (g/dL)	4.3 ± 0.6	4.2 ± 0.3	0.57
Uric acid (mg/dL)	7.7 ± 1.6	5.9 ± 1.6	0.07

Contrast-induced nephropathy (CIN) was defined as an increase in serum creatinine by at least 25% 48 h following contrast administration *vs*. baseline. Anemia was considered present when hemoglobin was < 13 g/dL and/or the hematocrit was < 39% in males, and when hemoglobin was < 12 g/dL and/or the hematocrit was < 36% in females, respectively.

Quantitative data are expressed as mean values ± standard deviation or median and interquartile range, as appropriate. Categorical data are expressed as number (percentage). p-values refer to comparisons between patients with and without CIN based on the unpaired *t*-test or the Mann–Whitney *U* test, as appropriate, for continuous variables, and Fisher’s exact test for categorical variables.

*ACEI* angiotensin converting enzyme inhibitor, *ARB* angiotensin II receptor blocker, *CIN* contrast-induced nephropathy, *eGFR* estimated glomerular filtration rate, *NYHA* New York Heart Association.

**Table 3:** Logistic regression analysis of predictors of contrast-induced nephropathy.

Parameter	OR (95%CI)	p-value
Left ventricular ejection fraction on admission ≤ 45%^a^	3.80 (0.24–58.84)	0.58
Diuretic therapy on admission	1.48 (0.27–88.53)	0.99
Anemia on admission	2.31 (0.11–45.61)	0.45
Volume of contrast agent administered > 180 mL^a^	4.78 (2.06–20.53)	0.001
Serum creatinine on admission > 1.62 mg/dL^a^	6.40 (2.50–25.20)	0.03

Contrast-induced nephropathy was defined as an increase in serum creatinine by at least 25% 48 h following contrast administration *vs*. baseline. Anemia was considered present when hemoglobin was < 13 g/dL and/or the hematocrit was < 39% in males, and when hemoglobin was < 12 g/dL and/or the hematocrit was < 36% in females, respectively.

^a^Cutoff values for left ventricular ejection fraction on admission, volume of contrast agent administered, and serum creatinine on admission were established using receiver operating characteristic analysis.

Early subclinical kidney injury, defined as ≥ 25% increase in NGAL levels at 48 h after contrast administration compared to baseline, was identified in 32 (17.9%) of the 178 study patients. Only 3 of these 32 patients presented CIN, defined as ≥ 25% increase in serum creatinine levels 48 h after contrast administration compared to baseline, whereas in the other patients there was no significant change in serum creatinine (p = 0.32). One month following contrast administration, NGAL levels remained stationary (79.75 ± 31.91 ng/mL *vs.* 76.64 ± 39.64 ng/mL, p = 0.14) and significantly positively correlated (r = 0.73, 95%CI 0.61–0.86; p < 0.01) with those measured 48 h after contrast administration. Subclinical kidney injury was still present 1 month after contrast administration in 17 (26.9%) of the 63 patients that underwent the 1-month follow-up. All those 17 patients were among the 32 patients who presented early subclinical kidney injury (53.1%); in 9 (28.1%) of those 32 patients the renal injury regressed after 1 month, and 6 (18.7%) of those patients were lost of follow-up.

Compared to patients who did not develop early subclinical kidney injury, those who presented an increase in NGAL levels ≥ 25% 48 h after contrast administration *vs.* baseline (Table 4) had higher serum creatinine and lower eGFR on admission (both p < 0.01), and higher total protein levels (both on admission [p = 0.04] and at the 48-h follow-up [p = 0.001]), had higher Mehran scores (p = 0.02) and lower sodium levels at the 48 h follow-up (p = 0.02), but there was no significant between-groups difference in the volume of contrast media administered (p = 0.67). In the logistic regression analysis, a baseline serum creatinine > 1.37 mg/dL (and a baseline eGFR ≤ 54 mL/min/1.73 m^2^; Supplementary material) remained the only independent predictor of early subclinical kidney injury (Table 5). None of the tested parameters was significantly different between patients who presented and those who did not present late subclinical kidney injury at the 1-month follow-up (all p > 0.05).

**Table 4:** Characteristics of patients who developed *versus* those who did not develop early subclinical kidney injury.

Parameter	Early injury (n = 32)	No early injury (n = 146)	p-value
**Baseline characteristics**			
Age (years)	65.5 ± 10.3	63.5 ± 9.2	0.51
Male gender (n, %)	24 (75.0%)	99 (67.8%)	0.52
Body mass index (kg/m^2^)	29.9 ± 3.4	27.9 ± 3.2	0.10
Left ventricular ejection fraction on admission (%)	49.1 ± 11.2	50.8 ± 9.2	0.73
Comorbidities			
Arterial hypertension (n, %)	29 (90.6%)	133 (91.0%)	1.00
Diabetes mellitus (n, %)	16 (50.0%)	64 (43.8%)	0.56
Heart failure (NYHA class)	2 (1.5–2.0)	2 (2.0–2.0)	0.60
Ongoing therapy with potentially nephrotoxic drugs			
ACEI (n, %)	21 (65.6%)	95 (65.0%)	1.00
ARB (n, %)	11 (34.3%)	28 (19.1%)	0.09
Diuretic (n, %)	19 (59.3%)	61 (41.7%)	0.07
Metformin (n, %)	11 (34.3%)	32 (21.9%)	0.17
Ongoing therapy with potentially nephroprotective drugs			
Statin (n, %)	31 (96.8%)	145 (99.3%)	0.32
Dihydropyridine calcium channel blocker (n, %)	13 (40.6%)	60 (41.0%)	1.00
Inferior vena cava diameter (cm)	1.9 ± 0.1	2.0 ± 0.1	0.42
Laboratory parameters on admission			
White blood cells (/mm^3^)	7525 ± 2052	7653 ± 1915	0.89
Platelets (/mm^3^)	230,583 ± 73,775	227,698 ± 66,697	0.89
Hemoglobin (g/dL)	13.6 ± 1.6	13.3 ± 1.6	0.52
Anemia (n, %)	11 (34.3%)	42 (28.7%)	0.52
Glucose (mg/dL)	147.9 ± 70.8	141.5 ± 63.6	0.88
Total cholesterol (mg/dL)	163.7 ± 53.6	162.5 ± 49.6	0.92
Triglycerides (mg/dL)	146.4 ± 55.2	162.3 ± 112.9	0.98
Total protein (g/dL)	68.7 ± 5.9	65.6 ± 4.3	0.04
Albumin (g/dL)	4.3 ± 0.3	4.3 ± 0.2	0.94
Uric acid (mg/dL)	6.3 ± 1.6	6.1 ± 1.7	0.73
Serum creatinine (mg/dL)	1.30 ± 0.25	1.00 ± 0.24	< 0.01
eGFR (mL/min/1.73 m^2^)	66.58 ± 23.50	77.40 ± 19.49	< 0.01
Pre-procedural intravenous hydration (n, %)	9 (28.1%)	26 (17.8%)	0.21
Contrast agent administered			
Type-iomeprol (n, %)	29 (90.6%)	124 (84.9%)	0.57
Volume (mL)	153.3 ± 88.8	149.2 ± 62.1	0.67
**Parameters at 48 h after contrast administration**			
Mehran score (points)	6 (3.5–8.0)	4 (1.0–6.0)	0.02
Therapy with potentially nephrotoxic drugs during the past 48 h			
ACEI (n, %)	21 (65.6%)	93 (63.6%)	1.00
ARB (n, %)	11 (34.3%)	32 (21.9%)	0.17
Diuretic (n, %)	19 (59.3%)	65 (44.5%)	0.17
Metformin (n, %)	8 (25.0%)	35 (23.9%)	1.00
Therapy with potentially nephroprotective drugs during the past 48 h			
Statin (n, %)	31 (96.8%)	145 (99.3%)	0.32
Dihydropyridine calcium channel blocker (n, %)	13 (40.6%)	67 (45.8%)	0.69
Length of hospital stay (days)	5.3 ± 3.0	4.9 ± 2.4	0.79
Laboratory parameters 48 h after contrast administration			
Sodium (mEq/L)	139.7 ± 1.7	141.5 ± 2.2	0.02
Total protein (g/dL)	69.6 ± 5.8	64.6 ± 4.4	0.001
Albumin (g/dL)	4.4 ± 0.4	4.2 ± 0.3	0.30
Uric acid (mg/dL)	6.5 ± 1.9	5.9 ± 1.6	0.22

Early subclinical kidney injury (early injury) was defined as an increase in NGAL by at least 25% 48 h following contrast administration *vs*. baseline. Anemia was considered present when hemoglobin was < 13 g/dL and/or the hematocrit was < 39% in males, and when hemoglobin was < 12 g/dL and/or the hematocrit was < 36% in females, respectively.

Quantitative data are expressed as mean values ± standard deviation or median and interquartile range, as appropriate. Categorical data are expressed as number (percentage). p-values refer to comparisons between patients with and without early subclinical kidney injury based on the unpaired *t*-test or the Mann–Whitney *U* test, as appropriate, for continuous variables, and Fisher’s exact test for categorical variables.

*ACEI* angiotensin converting enzyme inhibitor, *ARB* angiotensin II receptor blocker, *eGFR* estimated glomerular filtration rate, *NYHA* New York Heart Association.

**Table 5:** Logistic regression analysis of predictors of early subclinical kidney injury.

Parameter*	OR (95%CI)	p-value
Total protein on admission > 68.2 g/dL	5.68 (0.87–36.76)	0.06
Mehran score > 4	4.22 (0.25–15.37)	0.12
Sodium level at the 48-h follow-up < 140 mEq/L	2.50 (0.38–16.18)	0.33
Serum creatinine on admission > 1.37 mg/dL	10.92 (5.27–13.64)	0.02

Early subclinical kidney injury was defined as an increase in NGAL by at least 25% 48 h following contrast administration vs. baseline.

*Cutoff values for the evaluated parameters were established using receiver operating characteristic analysis.

## Discussion

**Figure 1:**
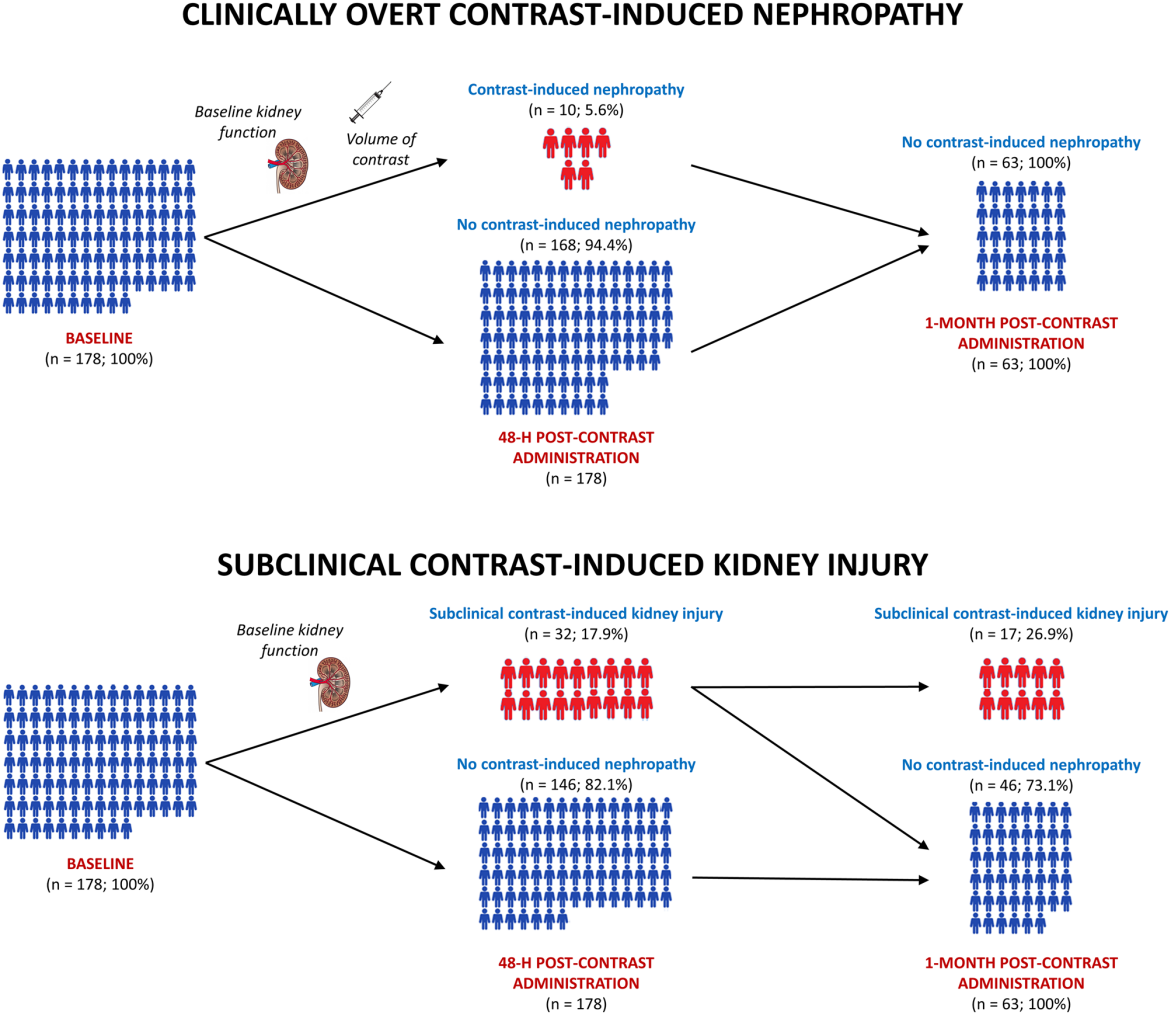
Overview of the main study findings.

The main findings of the present study (Fig. 1) were that (1) clinically overt CIN was rather rare, affecting 5.6% of the study population, (2) its occurrence was only affected by the baseline kidney function and the volume of contrast administered, and that (3) it was entirely regressive at the 1-month follow-up. Meanwhile, (4) subclinical kidney injury was considerably more common, affecting almost 18% of the study patients at the 48-h follow-up, (*5*) was independently predicted only by the baseline kidney function, and (6) was persistent at the 1-month follow-up in more than 50% of the initially affected patients.

### In the era of modern iodinated contrast media, clinically overt contrast-induced nephropathy is rather rare and entirely regressive after 1 month

With the progressive increase in the prevalence of atherosclerotic disease, the rate of angioplasty procedures is on a continuous rise worldwide^1^. One of the most feared complications of such procedures is CIN. However, our data indicate that in the era of modern iodinated contrast agents, the incidence of CIN is rather low, affecting 5.6% of the study patients.

Several large-scale studies have even questioned the concept of CIN and the relationship between contrast media administration and acute kidney injury^10^. Indeed, studies have shown similar rates of acute kidney injury in patients undergoing computed tomography scanning with and without intravenous administration of contrast media, and suggested that the risk of CIN may essentially be nonexistent in patients with normal baseline renal function^11^. The relationship between contrast media administration and CIN appears to be, however, rather solid, when the contrast agent is administered intraarterially^12^. Several hypotheses have been proposed to explain this discordance. Patients that undergo angiography procedures have more severe arterial disease than those who require intravenous administration of contrast media and are therefore at higher risk of acute kidney injury. Manipulation of the aorta could lead to dislodgement of cholesterol crystals, atherosclerotic plaque fragments, or thrombi, and to renal microembolization and could therefore promote kidney injury during intraarterial procedures^13^. Moreover, intraarterial administration of contrast media, particularly in the abdominal aorta, above the level of the renal arteries, is associated with higher contrast concentrations in the renal vasculature^12^. Together, these data suggest that the risk of CIN is therefore not directly linked to the route of contrast administration, but rather to the patients’ comorbid conditions, the characteristics of the procedure, and the volumes of contrast media administered.

Indeed, several risk factors have been associated with an increased risk of CIN. Among them, preexisting renal disease has been proposed as the most relevant risk factor for CIN. A linear relationship has been shown to exist between baseline serum creatinine and the risk of CIN, with an incidence of contrast-induced acute kidney injury of up to 62% in patients with preexisting chronic kidney disease and serum creatinine ≥ 2 mg/dL^14^. Due to the major impact that diabetes mellitus exhibits on the renal and cardiovascular systems^15^, this condition was also seen as a major non-modifiable risk factor for CIN^16^. Several other parameters, such as advanced age^16^, female gender^17^, history of congestive heart failure^18^, anemia^19^, hyperuricemia^20^, hypercholesterolemia^14^, and the use of nephrotoxic drugs^21^, have also been linked to an increased risk of CIN in various clinical studies, although their impact remains highly controversial^12^. Multiple sources have also reported a dose-dependent relationship between the volume of contrast administered and the risk of CIN^22^. In the present study, only basal renal function, as reflected by the baseline serum creatinine, and an increased volume of contrast administered were independently associated with an increased risk of CIN. For other parameters that were associated with CIN in univariate analysis (i.e., left ventricular ejection fraction, ongoing diuretic treatment, and anemia), the association was lost in the multiple logistic regression analysis.

In accordance with previous studies^23^, clinically overt CIN was a transient, reversible event in the present study, and was associated with more prolonged hospital stay.

### Subclinical kidney injury is rather common after administration of iodinated contrast agents and persists in more than half of patients at 1-month follow-up

Clinically overt renal dysfunction related to contrast administration therefore appears to be entirely regressive in one month. However, mechanistically, it seems unlikely that contrast-induced renal injury could be fully devoid of any long-term impact. The pathophysiology of CIN remains incompletely elucidated at this point. The most accepted theory involves contrast-induced vasoconstriction, leading to renal hypoxia^24^, increased production of oxygen-free species, and subsequently to renal injury^25^. Other factors, including a rise in blood viscosity, changes induced by the contrast media on the renal blood supply^26^, ischemia–reperfusion injury, release of angiotensin II, dopamine, and vasopressin, and a direct cytotoxic effect of contrast agents on the renal tubular cells have also been shown to contribute to the deleterious effects exhibited by contrast media on the kidneys^16^. These mechanisms strongly suggest that contrast agents may not be entirely innocuous over the long term and that, similar to other clinical settings^27,28^, measurement of biomarkers more sensitive than serum creatinine may be required to detect subtle renal changes in this setting. The diagnosis of CIN relies at present on measurement of serum creatinine. This approach has, however, several limitations, including the delayed and non-linear response to renal impairment of serum creatinine and its sensitivity to numerous non-renal factors, such as age, gender, diet, medication, muscle mass, hydration status, and volume of intravascular fluid^29^. Moreover, creatinine is a marker of glomerular filtration, and not a marker of tubular damage, which is the injury typical for CIN. Meanwhile, NGAL has been proposed as one of the most promising biomarkers of renal structural injury^30^. Unlike serum creatinine, NGAL is produced by the distal nephron and rapidly released into the bloodstream, which makes NGAL a much more sensitive marker of kidney injury^31^. Studies have also pointed NGAL as an earlier marker of kidney injury than serum creatinine in various clinical settings, including in patients with normal renal function, with septic shock, or post-cardiac surgery^4,32,33^, causing NGAL to be seen as a ‘kidney troponin’^34^. Based on these data, the Acute Dialysis Quality Initiative proposed a combination of kidney functional (i.e., serum creatinine) and structural (e.g., NGAL) damage markers to stratify the risk of acute kidney damage^30^.

In line with these data, in the present study, repeated NGAL evaluation demonstrated that acute renal injury was much more common than reflected by serum creatinine, affecting almost 18% of the study patients. Moreover, our data indicate that unlike CIN, which was regressive at the 1-month follow-up, subclinical kidney injury was still present after 1 month in more than half of patients in whom the kidneys were initially affected by the contrast media. In addition, similarly to what was seen for clinically overt CIN, the occurrence of subclinical kidney injury was also independently associated in the present study with the basal renal function, as reflected by the baseline serum creatinine. Moreover, the risk of developing subclinical kidney injury following contrast administration was related to a lower degree of baseline kidney dysfunction than the risk of clinically overt CIN.

### Clinical implications

In line with previous studies, our data indicate that the occurrence of clinically overt CIN is favored not only by non-modifiable (baseline kidney function), but also by modifiable factors—the volume of contrast administered. Technological innovations and technical adjustments, such as lowering the X-ray voltage or using the latest generation imaging platforms could thus be of use for CIN prevention by reducing the amount of contrast media that is being administered^35^. However, such approaches may not be efficient for reducing the risk of contrast-induced subclinical kidney injury. Unlike CIN, contrast-induced subclinical kidney injury was not affected by contrast volume in the present study, but only by a non-modifiable factor—the baseline kidney function. Oral and intravenous hydration, and pharmacologic strategies such as methylxanthines, statins, ascorbic acid, N-acetyl cysteine, or dihydropyridine calcium channel blockers have all been proposed as potentially efficient interventions for the prevention of CIN^16^. None of the patients included in the present study was receiving methylxanthines, ascorbic acid, or N-acetyl cysteine, and almost all patients (i.e., 98.8%) were undergoing statin therapy. Thus, the potential impact of such strategies on the risk of CIN could not be evaluated in the present study. Dihydropyridine calcium channel blockers did not appear to affect, however, the risk of CIN or of subclinical kidney injury in the present study. Previous studies have also suggested a higher risk of CIN with low-osmolar than with high-osmolar contrast media in patients with pre-existing chronic kidney disease^36^, whereas the benefit of iso-osmolar over low-osmolar contrast agents remains debatable^37,38^. In the present study, there was no significant difference in the type of contrast media used between patients with and without CIN or subclinical kidney injury. However, all patients in this study received low-osmolar contrast agents and more than 85% of them received the same contrast agent (i.e., iomeprol).

### Strengths and limitations

The effects of contrast media at the renal level were assessed in a prospective study, using both functional (i.e., serum creatinine) and structural (i.e., NGAL) renal damage markers, providing a comprehensive view on contrast-induced kidney injury. In addition, to the best of our knowledge, this is the first study to evaluate the long-term effects of contrast media on subclinical kidney injury, as reflected by the levels of NGAL. These analyses demonstrated that subclinical kidney injury was still present after 1 month in more than half of patients in whom the kidneys were initially affected by the contrast media, suggesting that these patients may be at increased risk for further, potentially clinically significant renal impairment, particularly if exposed to nephrotoxic agents or repeated administration of contrast media. Studies with longer-term follow-up of renal function, including after repeated administration of contrast agents, will have to clarify this issue. The long-term impact of the renal changes identified in the present study on ‘hard’ clinical endpoints (e.g., dialysis, death) also remains to be clarified. In the present study, only 35.3% of the study patients underwent, however, the 1-month follow-up. The high rate of loss of follow-up was mainly related to the fact that the angioplasty procedures took place in a tertiary center, and the patients’ subsequent follow-ups were mainly performed in their home centers. Future studies will have to further elucidate the impact of contrast media on subclinical kidney injury over the long term. The prospective nature of the present study allowed us to evaluate the renal impact of a large series of parameters associated with contrast-induced kidney injury in previous studies. Yet, only baseline kidney function and the volume of contrast administered were identified as independent predictors of CIN, whereas subclinical contrast-induced kidney injury was only independently predicted by the baseline serum creatinine. The relatively low number of patients included in the present study may have affected our ability to detect other potential predictors of contrast-induced acute kidney injury. However, with the exception of baseline kidney function, which has been related to CIN in the vast majority of previous studies, the role of the other tested factors is highly controversial in the literature^12^. Although NGAL is clearly a valuable biomarker of contrast-induced subclinical kidney injury, one should be aware that NGAL is not specific for this condition and that low levels of NGAL can also originate from other sources, such as neutrophils, cardiomyocytes, prostatic cells, or respiratory and gastrointestinal epithelia^4^. None of the patients evaluated at the 1-month follow-up presented anamnesis, clinical signs or symptoms of inflammatory disease. However, no specific laboratory analyses were performed at that time to this end. To date, there is no official definition for early subclinical kidney injury. In the present study, subclinical kidney injury was defined by analogy with clinically overt CIN. Future long-term studies will have to establish the most appropriate NGAL cut-off values for the diagnosis of subclinical contrast-induced kidney injury. Finally, the impact of contrast media on the kidneys was evaluated in the present study using serum creatinine and NGAL. Evaluation of additional parameters, such as urinary NGAL, molecule-1, or cystatin C would also have been of interest to fully elucidate the renal effects of contrast media.

## Conclusions

The present study showed that in the era of modern contrast media, clinically overt CIN is rather rare, regressive, and that its occurrence is affected by only the baseline renal function and the amount of contrast media administered. Subclinical kidney injury was, however, considerably more frequent in patients receiving intraarterial contrast media. More importantly, subclinical contrast-induced kidney injury persisted after 1 month in more than 50% of the initially affected patients. Pending confirmation in future studies, these data suggest that patients who develop subclinical contrast-induced kidney injury may be at increased risk for further, potentially clinically significant renal impairment, particularly if exposed to nephrotoxic agents or repeated administration of contrast media.

## Supplementary Information

Supplementary Tables.

## Data Availability

The datasets used and analyzed during the current study are available from the corresponding author on reasonable request.

## Abbreviations

CIN: Contrast-induced nephropathy
eGFR: Estimated glomerular filtration rate
NGAL: Neutrophil gelatinase-associated lipocalin
