# Supplementary material for: Neutrophil gelatinase-associated lipocalin monitoring reveals persistent subclinical kidney injury following intraarterial administration of iodinated contrast agents
Source: Sci Rep. 2022 Nov 14;12:19464. doi: 10.1038/s41598-022-24169-7 (PMC9663446; doi:10.1038/s41598-022-24169-7)
Supplement: Supplementary file 1 — Supplementary Tables. [file 41598_2022_24169_MOESM1_ESM.docx]

**Supplementary material.**

**Logistic regression analysis of predictors of contrast-induced nephropathy.**

| **Parameter** | **OR (95%CI)** | **p-value** |
| --- | --- | --- |
| Left ventricular ejection fraction on admission ≤ 45%* | 4.01 (0.23-68.27) | 0.33 |
| Diuretic therapy on admission | 1.72 (0.12-72.14) | 0.99 |
| Anemia on admission | 3.06 (0.17-53.24) | 0.44 |
| Volume of contrast agent administered >180 mL* | 6.55 (1.79-34.36) | < 0.01 |
| eGFR on admission ≤ 63 mL/min/1.73 m^2^* | 7.75 (3.14-19.11) | 0.03 |

Contrast-induced nephropathy was defined as an increase in serum creatinine by at least 25% 48 hours following contrast administration *vs*. baseline. Anemia was considered present when hemoglobin was < 13 g/dL and/or the hematocrit was < 39% in males, and when hemoglobin was < 12 g/dL and/or the hematocrit was < 36% in females, respectively.

*Cutoff values for left ventricular ejection fraction on admission, volume of contrast agent administered, and eGFR on admission were established using receiver operating characteristic analysis.

eGFR – estimated glomerular filtration rate

**Logistic regression analysis of predictors of early subclinical kidney injury.**

| **Parameter*** | **OR (95%CI)** | **p-value** |
| --- | --- | --- |
| Total protein on admission >68.2 g/dL | 5.52 (0.85-28.07) | 0.09 |
| Mehran score >4 | 1.80 (0.33-9.81) | 0.49 |
| Sodium level at the 48-hours follow-up < 140 mEq/L | 1.36 (0.30-6.16) | 0.68 |
| eGFR on admission ≤ 54 mL/min/1.73 m^2^ | 8.30 (1.38-49.56) | 0.02 |

Early subclinical kidney injury was defined as an increase in NGAL by at least 25% 48 hours following contrast administration *vs*. baseline.

*Cutoff values for the evaluated parameters were established using receiver operating characteristic analysis.

eGFR – estimated glomerular filtration rate
